# Ratiometric Analysis of Fura Red by Flow Cytometry: A Technique for Monitoring Intracellular Calcium Flux in Primary Cell Subsets

**DOI:** 10.1371/journal.pone.0119532

**Published:** 2015-04-02

**Authors:** Emily R. Wendt, Helen Ferry, David R. Greaves, Satish Keshav

**Affiliations:** 1 Nuffield Department of Clinical Medicine, Experimental Medicine Division, Translational Gastroenterology Unit, University of Oxford, Oxford, United Kingdom; 2 Sir William Dunn School of Pathology, University of Oxford, Oxford, United Kingdom; Faculty of Medicine & Health Sciences, UNITED ARAB EMIRATES

## Abstract

Calcium flux is a rapid and sensitive measure of cell activation whose utility could be enhanced with better techniques for data extraction. We describe a technique to monitor calcium flux by flow cytometry, measuring Fura Red calcium dye by ratiometric analysis. This technique has several advantages: 1) using a single calcium dye provides an additional channel for surface marker characterization, 2) allows robust detection of calcium flux by minority cell populations within a heterogeneous population of primary T cells and monocytes 3) can measure total calcium flux and additionally, the proportion of responding cells, 4) can be applied to studying the effects of drug treatment, simultaneously stimulating and monitoring untreated and drug treated cells. Using chemokine receptor activation as an example, we highlight the utility of this assay, demonstrating that only cells expressing a specific chemokine receptor are activated by cognate chemokine ligand. Furthermore, we describe a technique for simultaneously stimulating and monitoring calcium flux in vehicle and drug treated cells, demonstrating the effects of the Gαi inhibitor, pertussis toxin (PTX), on chemokine stimulated calcium flux. The described real time calcium flux assay provides a robust platform for characterizing cell activation within primary cells, and offers a more accurate technique for studying the effect of drug treatment on receptor activation in a heterogeneous population of primary cells.

## Introduction

Increases in the concentration of cytosolic free calcium is a rapid event following leukocyte activation and one measurement commonly used to quantify receptor stimulation [1,2]. Flow cytometry based calcium analysis has the advantage of multiple parameter analysis, in for example, exclusion of non-viable cells and selective gating on discrete cell populations [3,4]. Numerous calcium indicator dyes are commercially available, including the UV-excitable, Indo-1, and dyes excited at longer wavelengths, including Fura Red and Fluo-3 [5,6]. Dyes excited by longer wavelengths utilize commonly available lasers, whereas not all flow cytometry machines are equipped with UV lasers, owing to their large size and considerable cost, therefore the use of Indo-1 is not always possible.

Ratiometric analysis (ratio of increasing signal over decreasing signal) is the preferred method for monitoring calcium flux as it corrects for artifactual changes in fluorescence due to variations in indicator dye loading, changes in equipment focus, and effects of fluorescent bleaching [7]. Indo-1 is compatible with ratiometric analysis, and combining Fura Red with Fluo-3 produces ratiometric results comparable to Indo-1 [8,9]. Fura Red dye when used alone, is also compatible with ratiometric analysis and has been described for detection by confocal microscopy; however measuring calcium flux in real time by flow cytometry has been limited, likely owing to a weaker signal than that generated when Fluo-3 and Fura Red are combined [6,10]. However there are several advantages to using Fura Red alone for ratiometric detection: savings in time and cost by titrating a single dye; using a single dye introduces fewer variations between assays due to differences in dye loading; and an additional channel is available for staining cell surface antigens. Therefore, in some experimental situations, such as when multiple surface marker characterization is desired, using Fura Red dye alone may be desired, and here we describe such a case: studying chemokine receptor activation in primary leukocytes, simultaneously monitoring responses among distinct cell populations.

Chemokines are a family of small soluble cytokines, best described for their chemotactic properties [11,12]. Chemokines bind to seven trans-membrane, G-protein coupled chemokine receptors expressed on the surface of leukocytes initiating rapid intracellular signaling, including calcium mobilization, cytoskeletal rearrangements, and ultimately directed cell migration [13]. Chemokine receptors are differentially expressed among cell types and following cell activation and therefore expression of a particular receptor is often confined to discrete cell populations [14]. In coordination with other cell surface molecules (e.g. integrins, selectins and adhesion molecules), chemokines orchestrate the recruitment of inflammatory cells during injury and inflammation [15], and therapies targeting specific chemokine receptors is an active area of investigation [16,17]. Therefore improving techniques for target validation (validating antibody specificity) and drug evaluation (measuring drug specificity and potency) is valuable.

Here we describe the ratiometric analysis of Fura Red calcium dye, monitoring calcium flux within primary human leukocytes measured by flow cytometry. We describe how this technique can be optimized for different flow cytometers, to identify channels available for surface marker characterization. Measuring chemokine stimulated calcium flux, we show that this technique can robustly detect calcium flux within minority cell populations; we demonstrate that only chemokine receptor expressing cells respond to cognate chemokine ligand, while an analysis of the entire bulk population could produce false negative results.

In a novel technique, we demonstrate how this method can be adapted to measure the effect of drug treatment, simultaneously stimulating and measuring calcium flux within untreated and drug treated primary cells. This technique circumvents challenges associated with technical variations and is therefore a more accurate assessment of a commonly measured cell activation parameter. Finally, we demonstrate in freshly isolated peripheral blood mononuclear cells (PBMC), that calcium flux in response to chemokine stimulation can be detected in both monocytes and T cells.

## Materials and Methods

### Peripheral blood mononuclear cell (PBMC) isolation

Human blood was collected in either EDTA-coated tubes (Vacuette, Grenier Bio-One, Austria) or from a buffy coat (National Blood Service, Oxford, UK). PBMC were isolated by density centrifugation using Ficoll-Paque PLUS (GE Healthcare, UK) to the manufacturers description. Ethical approval was obtained from the National Research Ethics Service (reference number 11/YH/0020), and informed written consent was given by all study participants.

### Expanding human T cells

PBMCs were depleted of monocytes by CD14^+^ positive bead selection, conducted to the manufacturer’s instructions (Miltenyi Biotec, Bergisch Gladbach, Germany). Remaining cells were added at 2x10^6^ cells/ml in Complete growth media (RPMI-1640 with 10% foetal bovine serum, 50 U/ml penicillin and 50 μg/ml streptomycin) supplemented with 100 U/ml recombinant human interleukin-2 (IL-2) to plate bound anti-CD3 and anti-CD28 (3 μg/ml each). After 72 hours, cells were transferred to a new T75 tissue culture flask and replaced with fresh Complete growth media supplemented with IL-2. Cells were used for experiments between days 5 and 7 from initial isolation.

### Loading cells with calcium indicator dye

Cells were resuspended at 1x10^7^ cells/ml in 37°C Hanks Balanced Salt Solution (HBSS; Invitrogen, Paisley, UK) with 1 μM Fura Red, AM (Invitrogen, Paisley, UK) unless specified otherwise and 0.01% Pluronic F127, and incubated in a 37°C water bath for 30 minutes. Cells were washed in HEPES Buffered Saline Solution (HBSS with 1 mM CaCl_2_, 0.5 mM MgCl_2_, 0.1% BSA, 10 mM HEPES) and resuspended to 1x10^7^ cells/ml in HEPES Buffered Saline Solution with viability dye, SYTOX Green (Life Technologies, Carlsbad, CA, USA; diluted 1:1,500). Before analysis by flow cytometry, cells were allowed to equilibrate for ≥10 minutes in a 37°C water bath.

### Ratiometric calcium flux measured by flow cytometry

All experiments were performed on an LSRII SORP (BD Bioscience, NJ, USA) equipped with the following lasers: Blue laser emitting at 188 nm, 25 mWatt; Green laser emitting at 532 nm, 150 mWatt; Red laser emitting at 642 nm, 40 mWatt; Violet laser emitting at 406 nm, 25 mWatt. Calibration was performed using CaliBRITE Beads (BD, CA, USA).

Stimulant was prepared at 10X the final desired concentration in HEPES Buffered Saline Solution. Background, non-specific calcium flux was recorded for 25 seconds; the sample was removed, 180 μl of cell suspension was transferred to 20 μl of 10X stimulant and immediately replaced for recording. Recording was continuous at a rate of 8,000–10,000 events/second for 120 seconds in total.

Ratiometric analysis of Fura Red was measured by excitation by the Violet laser (406 nm) and the Green laser (532 nm). Emission was detected by two different filter sets: increases in emission were monitored off the Violet laser (630LP and 660/20 BP), while a decrease in emission was detected off the Green laser (685LP and 710/50 BP). The ratiometric, ‘Fura Red Ratio’ was calculated as the increasing signal stimulated by the Violet laser over the decreasing signal stimulated by the Green laser (406 nm / 532 nm) using the Kinetics tool in FlowJo software version 9.3.3 (Tree Star Inc., OR, USA).

### CCR6^+^ cell calcium flux

Human T cells were stained with anti-CCR6 antibody (eBioscience, clone R6H1, diluted 1:150) in Staining Buffer (PBS with 0.1% Bovine Serum Albumin) at a density of 1x10^7^ cells/ml and incubated at 37°C for 15 minutes. Cells were washed and stained with anti-mouse IgG-APC secondary antibody (Biolegend, poly4053, diluted 1:200) and incubated at 37°C for 15 minutes. Cells were washed twice in HBSS and loaded with 1 μM Fura Red, AM as previously described.

### Pertussis toxin (PTX) treatment

Cells were incubated at 37°C for 1 hour in Complete media supplemented with 200 nM PTX or an equivalent concentration of ethanol (vehicle).

### Calcium flux assay ± drug treatment

Vehicle- and PTX-treated cells were separately stained with anti-CCR6, followed by anti-mouse IgG-APC as previously described. Cells were washed two times in Labelling Buffer. Vehicle-treated cells were stained with anti-CD3 APC-eFluor780 (eBioscience, clone UCHT1, Hatfield, UK) and PTX-treated cells were stained with anti-CD3 AlexaFluor700 (eBioscience, clone UCHT1) diluted in Labelling Buffer and incubated for 15 minutes at 37°C. Cells were washed three times in RT HBSS. An equivalent number (determined by Trypan Blue Stain) of vehicle- and PTX-treated cells were combined to a final density of 1x10^6^ cells/ml and loaded with 1 μM Fura Red, AM as previously described.

### Calcium flux in whole PBMC

Whole PBMC were treated with FcR Blocking Reagent (Miltenyi Biotec, Bergisch Gladbach, Germany) to the manufacturer’s recommendations, incubated for 10 minutes at RT. PBMC were stained with anti-CD3 (AlexaFluor700, Biologend, clone UCHT1, diluted 1:300), anti-CD14 (FITC, Biolegend clone M5E2, diluted 1:300), anti-CD16 (Biolegend, clone 3G8, diluted 1:300), and anti-CCR2 (Biolegend, clone K036C2, diluted 1:300) in Staining Buffer at a density of 1x10^7^ cells/ml for 15 minutes at 37°C. Cells were washed and loaded with 1 μM Fura Red, AM as previously described.

### Chemicals and reagents

Supplied by Sigma Aldrich (St Louis, MO, USA) unless otherwise specified. All cytokines and chemokines sourced from Peprotech (Rocky Hill, NJ, USA), reconstituted according to the manufacturer’s recommendations, and stored in aliquots at -80°C.

## Results

### Ratiometric analysis of Fura Red calcium dye

Ratiometric analysis of a calcium dye relies upon detecting an increasing fluorescence signal as calcium is released into the cell cytoplasm, in combination with a decreasing signal as fluorescence is quenched by cytoplasmic calcium. Although the manufacturer of Fura Red describes ratiometric capacity when used alone, few published data have used the dye in such a manner. We examined the ratiometric potential of Fura Red, using primary human T cells and detection on an LSRII SORP.

To determine the optimal concentration of dye, we performed a titration of Fura Red, AM loading primary human T cells using a range of concentrations recommended by the manufacturer (1–10 μM). Calcium flux was stimulated by the ionophore, ionomycin. Ionomycin mobilizes calcium stored in the endoplasmic reticulum in a Mn^2+^ dependent manner [18].

**Fig 1:**
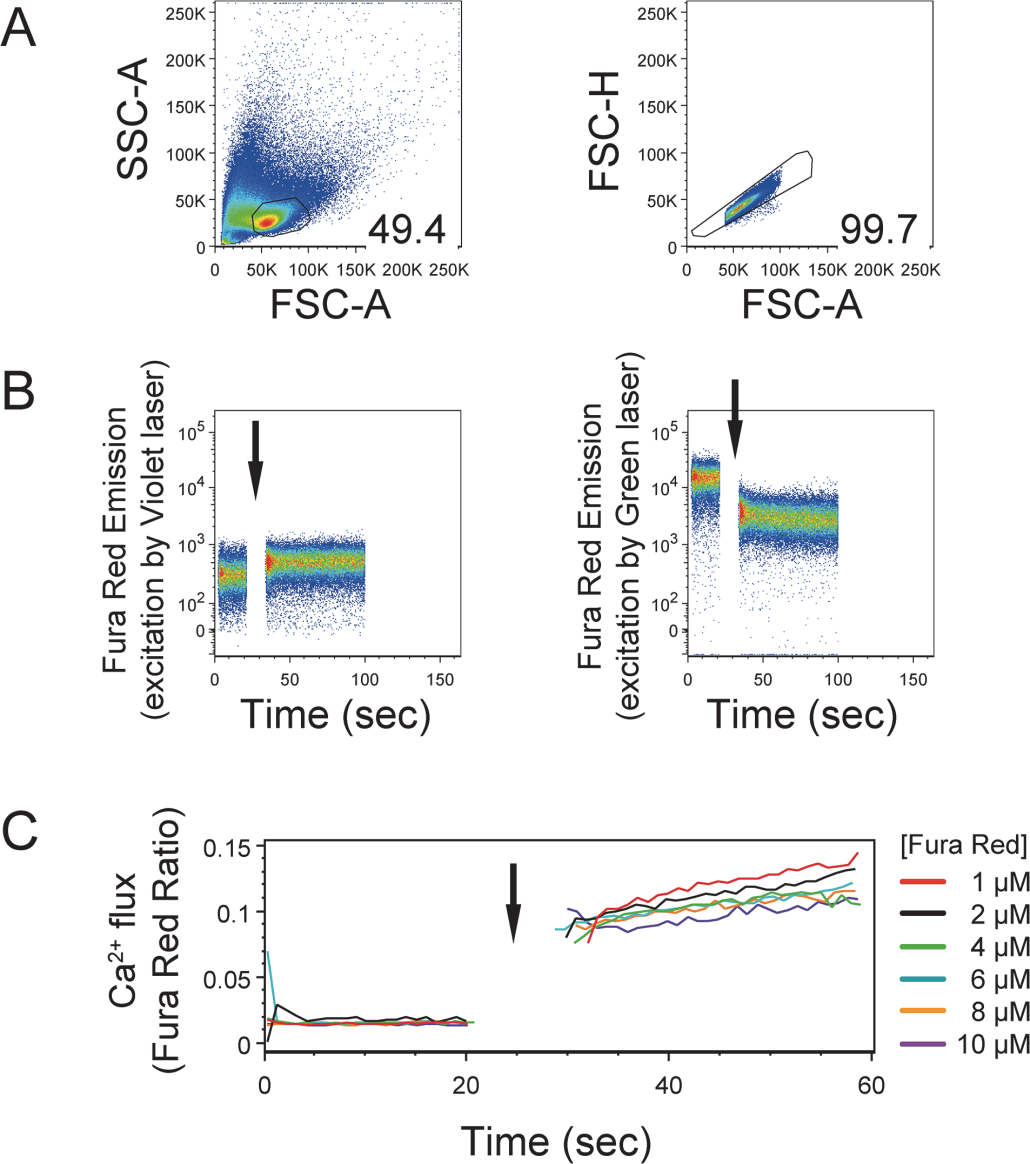
Ratiometric analysis of Fura Red calcium dye and Fura Red titration. Expanded human T cells were loaded with increasing concentrations of Fura Red, AM for 30 minutes. Intracellular calcium mobilization in response to ionomycin (5 μg/ml) was measured by flow cytometry. Arrows indicate the addition of ionomycin. (A) Gating parameters to select lymphocytes (based upon forward and side scatter properties) and singlets. (B) Intracellular calcium mobilization detected as a rise in fluorescence measured off the Violet laser (406 nm) and decreasing fluorescence measured off the Green laser (532nm). (C) Ratiometric analysis of Fura Red signal and dye titration. Fura Red Ratio calculated as emission off the Violet laser over emission off the Green laser. Values depict the mean of the ratio over time. One representative experiment of n = 2 shown.

By selective gating, lymphocytes were monitored, and cell doublets were excluded from analysis (Fig 1A). The single cell suspension was monitored for 20 seconds (background signal) to establish non-specific fluctuations in intracellular calcium. Following the addition of ionomycin, an increasing signal was detected off of the Violet laser (406 nm) and a concurrent decreasing signal was measured off the Green laser (532 nm) (Fig 1B). Ratiometric analysis of Fura Red dye was performed in FlowJo Software, using the Kinetics tool and calculated as 406nm/532nm (Fig 1C). Exposing cells to Fura Red and it’s diluent dimethylsulfoxide (DMSO) will cause cell death, therefore using low concentrations of the reagent is recommended. In the tested human T cells, there was no benefit to using greater than 1μM Fura Red, AM (Fig 1C).

### Identifying channels available for surface antigen detection in combination with Fura Red

Different flow cytometers will be equipped with different lasers and filters, making each configuration unique. Therefore to define channels available for surface characterization when cells are loaded with Fura Red dye, it is necessary to identify channels that are unaffected by changes to Fura Red emission following calcium flux.

**Fig 2:**
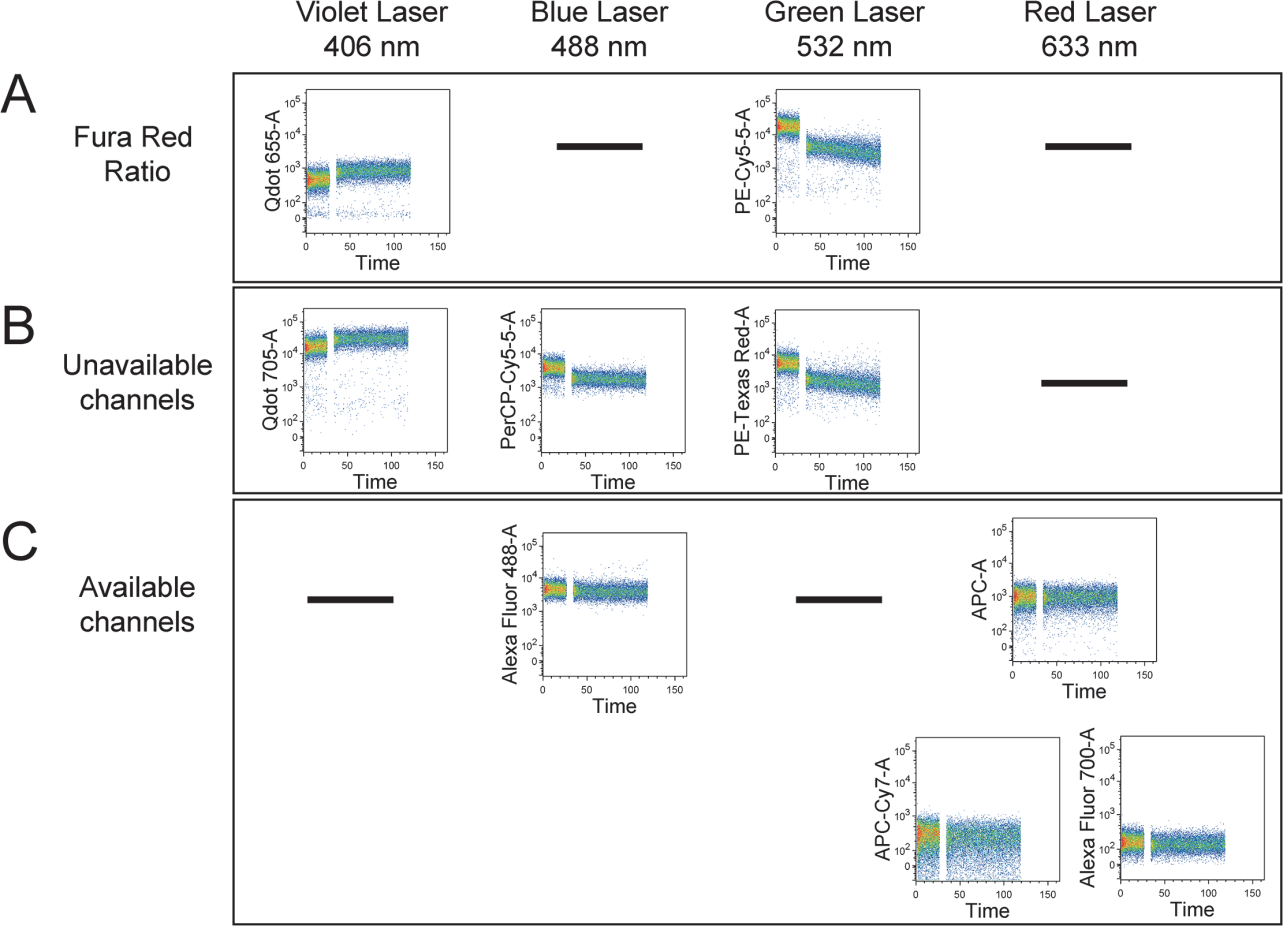
Identifying channels available in combination with Fura Red calcium dye. Expanded human T cells were loaded with 1 μM Fura Red, AM for 30 minutes. Calcium flux in response to ionomycin (5 μg/ml added at 25 seconds) was recorded on all available channels using an LSRII SORP flow cytometer. Cells were selectively gated on lymphocytes and singlets. The names assigned to the different detectors represent fluorochromes that are commonly detected in that channel. (A) Channels used to perform ratiometric analysis of Fura Red dye. (B) Representative examples of channels that display a shift in MFI following calcium mobilization. Channels such as these cannot be used for surface protein characterization in combination with Fura Red. (C) Channels with no change in MFI following calcium mobilization. Such channels are available for surface marker and cell viability analysis in combination with Fura Red dye. On the tested LSRII SORP, four available channels were identified, and fluorochromes commonly detected in these channels include: AlexFluor488, APC, APC-Cy7, and AlexaFluor700. One representative experiment of n = 2 shown.

Primary human T cells were loaded with Fura Red, AM, stimulated with ionomycin, and changes to mean fluorescence intensity (MFI) over time was measured on all available channels on an LSRII SORP. Ratiometric detection of Fura Red was monitored off the Violet and Green lasers, as advised by the manufacturer (Fig 2A). Fig 2B provides representative traces for channels that display shifts in MFI following calcium mobilization. These channels are therefore not suitable for surface antigen characterization. Channels that do not display a shift in MFI following calcium flux can be used for staining with a viability dye or for detecting surface antigens (Fig 2C). On the LSRII SORP, four available channels were identified, and fluorochromes commonly detected by these channels include: AlexaFluor488, APC, APC-Cy, and AlexaFluor700.

### Measuring calcium flux in discrete populations of primary cells

**Fig 3:**
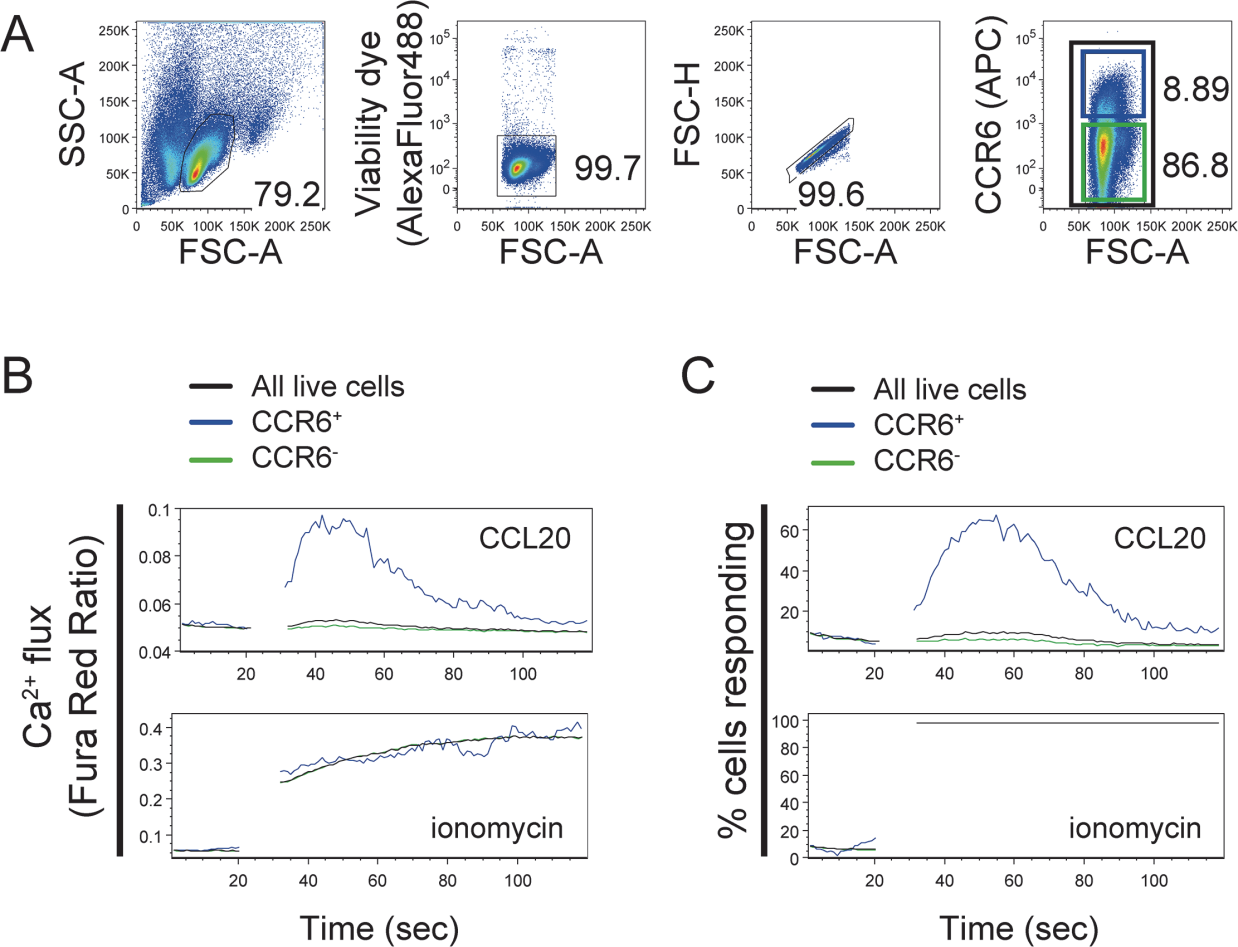
Analysis of calcium flux by discrete populations of primary cells. Expanded human T cells were stained for surface CCR6 protein, loaded with 1 μM Fura Red, AM and stained with a cell viability dye. Calcium flux in response to CCL20 (125 ng/ml) or ionomycin (5 μg/ml) was monitored by flow cytometry. (A) Gating strategy to exclude non-viable cells, select singlets, and distinguish CCR6^+^ from CCR6^-^ cells. (B) Calcium flux depicted as the mean value of the Fura Red Ratio over time, in response to CCL20 or ionomycin. (C) Calcium response depicted as the percent of cells responding to stimuli greater than the average background signal. One representative experiment of n = 5 shown, performed with a minimum of two technical replicates.

To demonstrate the utility of calcium flux monitored by flow cytometry, we used primary human T cells stained for CCR6, and monitored calcium flux in response to CCL20. CCL20 is the single ligand to CCR6, and a subset of peripheral blood T cells express surface CCR6 [19]. Fig 3A illustrates a gating strategy to exclude non-viable cells and cell doublets, and to selectively gate CCR6^+^ cells.

Human T cells expressing surface CCR6, and not CCR6^-^ cells, mobilized intracellular calcium in response to CCL20 (Fig 3B). An alternative analysis of the same data is to monitor the proportion of cells producing a calcium response (Fig 3C). Ionomycin stimulated a calcium response from both CCR6^+^ and CCR6^-^ T cells demonstrating that cells were loaded with an equivalent amount of Fura Red dye, had similar calcium stores available for mobilization, and the entire population of cells were capable of producing a calcium response (Fig 3B and 3C). Analysis of the entire live cell population (black line) produced a modest calcium signal relative to the selective gating technique (Fig 3B and 3C). These data illustrate that monitoring calcium flux within a heterogeneous cell population may lack the sensitivity to detect changes within minority cell populations.

### Simultaneously stimulating and detecting calcium flux in untreated- and drug-treated cells

Measuring the effect of drug treatment on calcium flux can be challenging when the effect size is small or the population of interest is a minority. Furthermore, variations between technical replicates can confound results, requiring many experimental replicates to confidently measure small effects. Here we describe a technique to differentially label vehicle- and drug-treated cells, allowing simultaneous stimulation and detection of real time calcium flux. In the presented example, the effect of Gαi inhibitor, Pertussis Toxin (PTX), is measured on human T cells expressing CCR6, in response to chemokine stimulation. Channels previously identified as available for surface marker characterization (refer to Fig 2C) were used to selectively label different cell populations.

**Fig 4:**
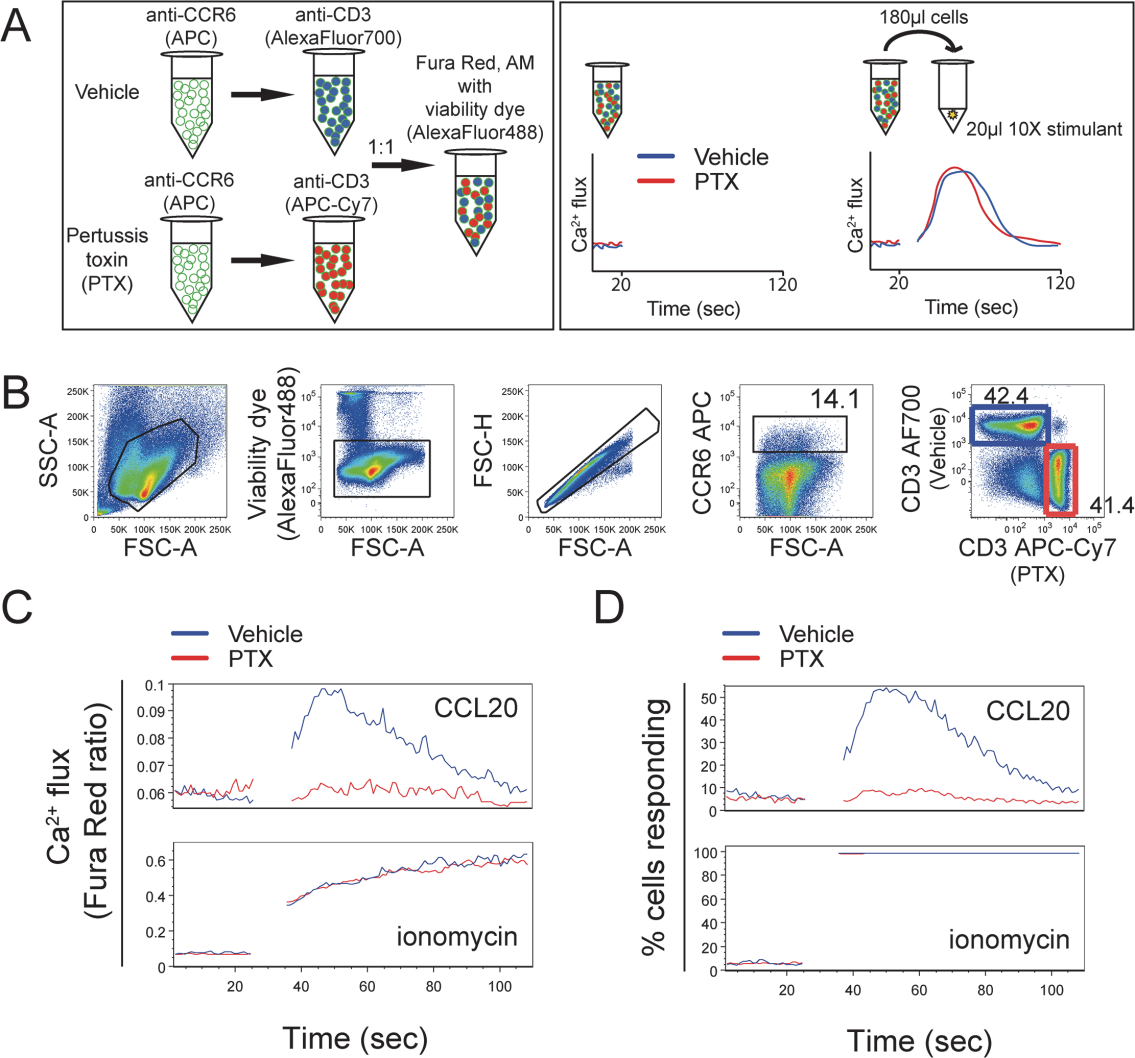
Calcium assay to simultaneously monitor vehicle- and drug-treated cells. Expanded human T cells were treated with vehicle (ethanol) or Gαi inhibitor, pertussis toxin (PTX) for 1 hour at 37°C. Vehicle and PTX treated cells were stained for surface antigens, loaded with 1 μM Fura Red, AM and stained with a cell viability dye. Chemokine stimulated calcium flux was monitored simultaneous in the vehicle and PTX groups by selective gating. (A) Schematic of staining protocol and data acquisition by flow cytometry. (B) Representative example of selective gating strategy to monitor live cells, singlets, CCR6^+^, distinguishing vehicle- and PTX-treated cells based on anti-CD3 antibodies conjugated to distinct fluorochromes (AlexaFluor700 or APC-Cy7). Numbers next to gated population indicate percentage of cells expressing antigen of interest. (C) Ratiometric analysis of Fura Red calcium dye depicted as mean Fura Red Ratio over time. Plots depict calcium flux by CCR6^+^ T cells in response to CCL20 (125 ng/ml), or ionomycin (5 μg/ml). Stimulus was added at 25 seconds. (D) Data from C analyzed as the proportion of cells producing a calcium signal greater than the average background signal. One representative experiment of n = 3 shown, performed with a minimum of two technical replicates.

Primary human T cells were treated with PTX for 1 hour; vehicle- and PTX-treated cells were separately labeled for CCR6 with antibody conjugated to APC. Vehicle- and PTX-treated cells were subsequently stained with anti-CD3 antibody conjugated to either APC-Cy7 or AlexaFluor700. The two cell populations were mixed in equal proportions and loaded with Fura Red, AM in the presence of the viability dye, SYTOX Green. Fig 4A illustrates the cell staining protocol and technique for data acquisition by flow cytometry.

By selective gating, live, singlet, CCR6^+^ T cells were monitored (Fig 4B). Although the proportion of CCR6^+^ cells was <15% of the total population, a robust calcium response to CCL20 was detected (Fig 4C and 4D). Among CCR6^+^ cells, vehicle- and PTX-treated CD3^+^ T cells could be distinguished by selective gating (Fig 4B). In primary human CCR6^+^ T cells, PTX-treatment reduced calcium mobilization in response to CCL20, quantified as the relative amount of mobilized calcium and the frequency of responding cells (Fig 4C and 4D). Ionomycin stimulation confirmed that vehicle and PTX-treated cells were evenly loaded with Fura Red dye, and capable of mounting an equivalent calcium response (Fig 4C and 4D). These data demonstrate that live, primary human T cells treated with PTX have an impaired response to CCL20 while untreated cells have a robust calcium response. By measuring the two populations simultaneously, we can be confident that the effects are not due to differences in technical replicates.

### Monitoring calcium flux in primary human PBMC

To determine if calcium flux can be measured in fresh PBMC, and in particular within monocytes, whole PBMC were isolated from fresh blood, and calcium flux in response to CCL2 was monitored. CCL2 (previously named MCP-1) is regarded as an inflammatory chemokine, produced at sites of inflammation. CCL2 binds CCR2, and CCR2 is expressed on monocytes, memory T cells and dendritic cells [20–22].

**Fig 5:**
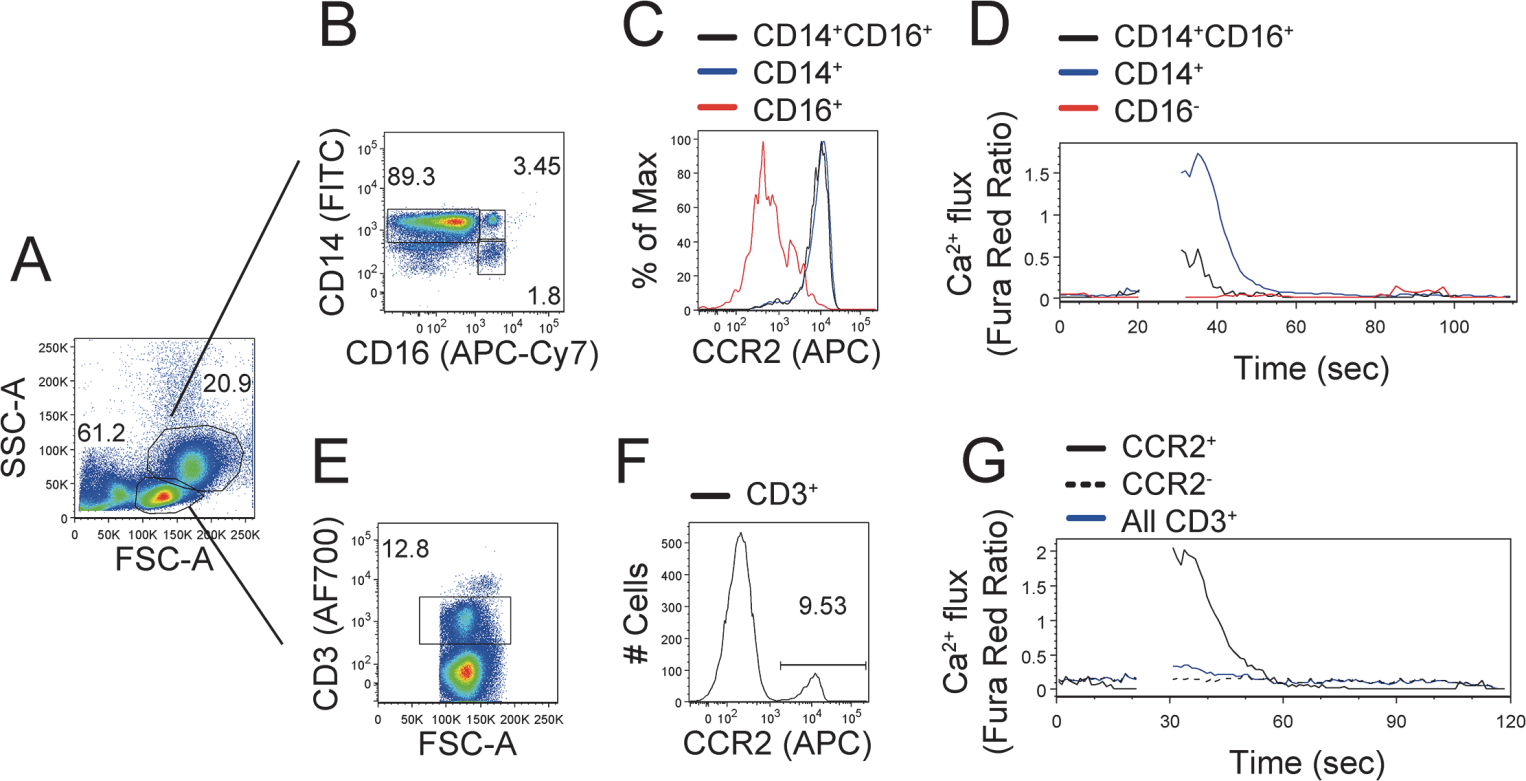
Calcium flux monitored in whole PBMC. Fresh PBMC were stained for surface markers CD3, CD14, CD16 and CCR2 and loaded with 1 μM Fura Red, AM. (A) Forward and side scatter properties were used to distinguish myeloid and lymphoid cells. (B) Monocyte subsets were distinguished on the basis of CD14 and CD16 expression. (C) Histogram depicts CCR2 expression by monocyte populations. (D) Real time calcium flux in response to CCL2 (65 ng/ml) added at 25 seconds, monitoring distinct monocyte populations. (E) T lymphocytes were identified by CD3 expression. (F) Histogram depicts CCR2 expression by T lymphocytes. (G) Real time calcium flux in response to CCL2 (65 ng/ml), monitoring CD3^+^ T lymphocyte subsets. The number next to selective gates indicates the proportion of cells among the parent population. One experiment of n = 4 shown, performed with three technical replicates.

Whole PBMC were labeled to detect CD3, CD14, CD16 and CCR2 (antibodies conjugated to AlexaFluor700, FITC, APC-Cy7 and APC, respectively) and loaded with Fura Red, AM. Calcium flux in response to CCL2 was monitored in real time. Peripheral blood monocytes have larger forward and side scatter properties than lymphocytes and can be further characterized by expression of surface CD14 (co-receptor for lipopolysaccharides) and CD16 (also known as FcγRIII). These two molecules are used to define two major monocyte subsets in peripheral blood, the ‘classical’ CD14^+^CD16^-^ and ‘non-classical’ CD14^low^CD16^+^[23,24]. Our staining could distinguish these populations (Fig 5A and 5B).

The majority of CD14^+^ and CD14^+^CD16^+^ cells express CCR2 and the majority of CD16^+^ cells are CCR2^-^ (Fig 5C). Following CCL2 stimulation, calcium flux was detected in the CCR2 expressing populations; interestingly, and potentially biologically relevant, CD14^+^ cells displayed a larger calcium response than the CD14^+^CD16^+^ cells. CD16^+^ cells did not respond to CCL2, corresponding with low expression of CCR2.

T lymphocytes were selectively gated by forward and side scatter properties, and by the expression of T cell receptor, CD3 (Fig 5A—5E). CCR2 expression was detected on a subset of T cells (Fig 5F). Calcium flux in response to CCL2 was detected among CCR2^+^ T cells and not by CCR2^-^ T cells (Fig 5G). These results demonstrate that real time intracellular calcium flux can be detected within primary monocyte and T cell subsets, simultaneously and without prior cell enrichment or expansion.

## Discussion

We describe the ratiometric analysis of calcium indicator dye, Fura Red monitored by flow cytometry. All of the described experiments were performed on an LSRII SORP with a configuration that allowed calcium flux detection in the presence of four additional fluorochromes. This technique however, can be adapted to any multiple color flow cytometer equipped with a Green and Violet laser. Indeed, we successfully performed similar experiments on an LSRFortessa (BD Bioscience, NJ, USA) although the particular configuration only allowed analysis of three additional fluorochromes (data not shown). We recommend that a preliminary experiment be conducted, as illustrated in Fig 2, to identify channels available for use in combination with Fura Red.

During optimization we identified several methods for improving the quality of data collected. Firstly, the more target cells available for analysis, the higher the quality of data produced. When using primary cells, this can be achieved by enriching the cell population of interest, either by cell sorting or by cell expansion, *in vitro*. Additionally, increasing the number of events acquired per second can improve data quality, and this can be achieved by either increasing the cell density prior to acquisition or by increasing the flow rate during acquisition on the flow cytometer.

The described technique has many practical applications. Rabin et al. (1999) demonstrated in human T cells, that chemokine receptor expression does not universally correlate to responsiveness to ligand [25]. Our described technique improves the ability to interrogate this relationship, with the potential for the analysis of multiple cell populations simultaneously. In our analysis of calcium flux to CCL2, measured in whole PBMC (distinguishing monocyte and T cell subsets), some blood donors had CCR2^+^ T cells that did not respond to CCL2 while in the same donor, monocytes displayed a robust calcium response. Profiling responding cell populations could improve our understanding of various diseases by scrutinizing both receptor expression and changes to receptor activation. In a healthy donor, approximately 1x10^6^ PBMC can be isolated per milliliter of fresh blood. From a 20ml blood donation, if 20x10^6^ PBMC are resuspended to 1x10^7^ cells/ml as described in the methods section, approximately 8 technical replicates can be tested. It is therefore feasible to study the calcium response of different blood donors and patient populations using fresh PBMC.

Another practical application we describe is measuring the effect of drug treatment on calcium signaling. By measuring calcium flux simultaneously in vehicle- and drug-treated cells, we circumvent problems associated with technical variations, and we reduce the number of cells and reagents needed. Furthermore, by analysing the treatment groups simultaneously, small changes can be more confidently interpreted. Along these lines, new drug candidates may display cytotoxicity; this technique allows non-viable cells to be excluded from analysis, improving the ability to measure an effect from drug treatment, independent from cytotoxicity.

In our analysis of CCL20 stimulation in primary expanded human T cells, we demonstrate that only CCR6^+^ cells responded to cognate ligand. Importantly, calcium flux by the entire population, where the proportion of CCR6^+^ target cells was low (<10%) displayed a relatively small response that could be falsely interpreted as a negative signal. The described technique has the distinct advantage of detecting a calcium response among a relatively small proportion cells. An additional application might be validating the specific and selectivity of antibodies, particularly when a minority population expresses the target of interest.

Measuring calcium flux by flow cytometry has been previously described, but to the best of our knowledge this is the first description of ratiometric detection of Fura Red in primary PBMC. The advantage to using Fura Red alone is that an additional channel is available for labeling surface antigens, only one dye needs to be purchased and titrated, and Fura Red utilizes lasers that are available on most flow cytometers. The described technique has several practical applications including measuring calcium flux in whole PBMC, independent of cell isolation or enrichment.
